# Differential fine-tuning of gene expression regulation in coffee leaves by *CcDREB1D* promoter haplotypes under water deficit

**DOI:** 10.1093/jxb/erx166

**Published:** 2017-07-11

**Authors:** Gabriel Sergio Costa Alves, Luana Ferreira Torres, Eveline Déchamp, Jean-Christophe Breitler, Thierry Joët, Frédéric Gatineau, Alan Carvalho Andrade, Benoît Bertrand, Pierre Marraccini, Hervé Etienne

**Affiliations:** 1 EMBRAPA Recursos Genéticos e Biotecnologia (LGM), Parque EB, Brasilia, DF, Brazil; 2 CIRAD, UMR IPME, F-34394 Montpellier, France; 3 Universidade Federal de Lavras, Departamento de Química, Laboratório Central de Biologia Molecular (LCBM), Lavras, MG, Brazil; 4 IRD, UMR DIADE, 911 Avenue Agropolis, Montpellier, France; 5 CIRAD, UMR AGAP, F-34398 Montpellier, France; 6 Embrapa Café, INOVACAFÉ, Campus UFLA, Lavras, MG, Brazil

**Keywords:** Coffee, DREB1D gene, genetic transformation, promoter haplotypes, uidA reporter gene, water deficit

## Abstract

Despite the importance of the *DREB1D* gene (also known as *CBF4*) in plant responses to water deficit and cold stress, studies analysing its regulation by transgenic approaches are lacking. In the current work, a functional study of three *CcDREB1D* promoter haplotypes (named HP15, HP16 and HP17) isolated from drought-tolerant and drought-sensitive clones of *Coffea canephora* was carried out in plants of *C. arabica* stably transformed by *Agrobacterium tumefaciens* by analysing their ability to regulate the expression of the *uidA* reporter gene in response to water deficit mimicked by polyethylene glycol (−2.0 MPa) and low relative humidity treatments. A deletion analysis of their corresponding 5′-upstream regions revealed increased specificity of β-glucuronidase activity in the polyethylene glycol and low relative humidity treatments, with high expression in leaf mesophyll and guard cells in full-length constructs. RT-qPCR assays also revealed that the HP16 haplotype (specific to clone tolerant to water deficit) had stronger and earlier activity compared with the HP15 and HP17 haplotypes. As most of the *cis*-regulatory elements involved in ABA-dependent and -independent networks, tissue specificity and light regulation are common to these haplotypes, we propose that their organization, as well as the nucleic acid polymorphisms present outside these boxes, may play a role in modulating activities of *DREB1D* promoters in guard cells.

## Teaser

Fine-tuning of *DREB1D* expression in stomatal guard cells under water deficit is mediated differentially by promoter haplotypes from sensitive and tolerant coffee genotypes.

## Introduction

Climate change is leading to increasingly extreme temperatures and drought periods, which are major abiotic factors affecting coffee production (DaMatta and Ramalho, 2006). Recent modelling studies have delivered warnings on the threat of severe droughts and high temperatures to coffee production by increasing attacks by pests and pathogens (Avelino *et al.*, 2004; Jaramillo *et al.*, 2011; Magrach and Ghazoul, 2015). These climatic events, which already affect coffee production, are expected to change the areas suitable for coffee cultivation (Davis *et al.*, 2012; Bunn *et al.*, 2015; Craparo *et al.*, 2015). Drought is a limiting factor that affects flowering and yield of coffee (DaMatta and Ramalho, 2006), as well as bean development and biochemical composition and hence the final quality of the beverage (Silva *et al.*, 2005; Vinecky *et al.*, 2016). Increased [CO_2_] in air is also a key factor for coffee plant acclimation to high temperature, strengthening the photosynthetic pathway, metabolism and antioxidant protection, and modifying gene transcription and mineral balance (Ramalho *et al.*, 2013; Martins *et al.*, 2014, 2016; Ghini *et al.*, 2015; Rodrigues *et al.*, 2016). In this context, understanding the genetic determinism of coffee’s adaptation to climate change has become essential for creating new varieties (Cheserek and Gichimu, 2012; van der Vossen *et al.*, 2015).

Within the *Coffea* genus, substantial genetic variability exists in relation to drought tolerance, particularly in the cultivated species *C. canephora* (Montagnon, 2000). This species, also commercially known as ‘Robusta’, is diploid (2*n*=2*x*=22), autogamous and phylogenetically separated into distinct groups according to geographical origin in the inter-tropical region of Africa (Cubry *et al.*, 2013). For instance, the Congolese group contains subgroup 1 (SG1), originating from the coastal lowlands and relatively tolerant to water deficit, while plants belonging to subgroup 2 (SG2) are more sensitive to water deficit. In Brazil, breeding programs implemented in the last decade on *C. canephora* Conilon (related to SG1, Montagnon *et al.*, 2012) identified several drought-tolerant (D^T^) and drought-sensitive (D^S^) clones, which were intensively characterized (Lima *et al.*, 2002; Pinheiro *et al.*, 2004; Praxedes *et al.* 2006). Among their anatomical and physiological traits, better root development and greater water use efficiency were highlighted as important factors of tolerance to water deficit in D^T^ clones (Pinheiro *et al.*, 2005).

In order to investigate the molecular determinism of tolerance to water deficit in coffee, several of these *C. canephora* clones (such as D^T^ clone 14 and D^S^ clone 22) were used to identify candidate genes whose expression was induced by drought stress (Marraccini *et al.*, 2011, 2012; Vieira *et al.*, 2013). Of those genes, *CcDREB1D* (encoding dehydration responsive element binding transcription factor 1D) was of outstanding interest since its expression was up-regulated under conditions of water deficit in leaves of D^T^ clone 14 but not in those of D^S^ clone 22 (Marraccini *et al.*, 2012; Vieira *et al.*, 2013).

The *DREB* gene family includes key transcription factors involved in responses to various abiotic stresses, such as water deficit, cold and salt stress, regulating the expression of essential responsive genes (Lata and Prasad, 2011; Sakuma *et al.*, 2002). DREB factors act downstream of ABA-dependent and -independent signal transduction pathways in abiotic stress responses. Indeed, the overexpression of *DREB* genes in several genetically engineered plants leads to up-regulation of cold-regulated genes and osmotic stress-responsive genes, resulting in increased abiotic stress tolerance (Agarwal *et al.*, 2006*b*). Several DREB transcription factors have also been functionally characterized in model plants. In Arabidopsis and rice, for instance, overexpression of *DREB1* and *DREB2* have shown promising results in inducing tolerance to cold, salt and water deficit (Novillo *et al.*, 2004; Ito *et al.*, 2006; Lim *et al.*, 2007; Kim *et al.*, 2011). However, *DREB* genes have been less extensively characterized in non-model (e.g. perennial) plants, particularly due to laborious genetic transformation protocols often limiting plant regeneration.

Even though *DREB1*/*CBF* (cold-binding factor) gene expression is mainly induced by cold, it was also reported to be involved in the control of plant development and response to other stress stimuli (Lata and Prasad, 2011). The expression of *AtDREB1D* (also known as *AtCBF4*) is rapidly induced by water deficit but not by cold in Arabidopsis (Haake *et al.*, 2002). Expression of the *CBF4* gene was also reported to be up-regulated in response to low temperatures, water deficit, and salinity in different *Vitis* species (Xiao *et al.*, 2008; Zandkarimi *et al.*, 2015) and in *Medicago truncatula* (Li *et al.*, 2011). The fact that overexpression of the *DREB1D*/*CBF4* gene increased tolerance to water deficit in transgenic plants of Arabidopsis (Haake *et al.*, 2002) and *Glycine max* (Guttikonda *et al.*, 2014) suggests that it plays an important role in plant responses to abiotic stress.

Despite the importance of *DREB* genes in plant response pathways to abiotic stress, a limited number of studies have analysed the regulation of their corresponding promoters. Promoter regions of *AtDREB1C* (Zarka *et al.*, 2003), *OsDREB1B* (Gutha and Reddy, 2008), *GmDREB3* (Chen *et al.*, 2009), and *AtDREB2C* (Chen *et al.*, 2012) were shown to be sufficient for regulating the transcription of the *uidA* reporter gene by abiotic stresses in transgenic Arabidopsis. More recently, Fang *et al.* (2015) also demonstrated that 1278 bp of the *FeDREB1* promoter from the common buckwheat enhanced β-glucuronidase (GUS) activities in drought in leaves of transgenic tobacco. However, functional characterization of the *DREB1D* promoter has not been reported. In an attempt to better understand the tissular and environmental regulation of *CcDREB1D* promoter activity, as well as the regulation of allele-specific expression depending on the haplotype sequence, our study set out to (i) isolate and compare the promoter haplotypes of *CcDREB1D* genes from D^T^ clone 14 and D^S^ clone 22 of *C. canephora*, (ii) evaluate their ability to regulate expression of the *uidA* reporter gene by monitoring GUS histochemical activity in transgenic plants of *C. arabica* subjected to different abiotic stresses, and (iii) analyse the expression of *uidA* and endogenous *CaDREB1D* genes in leaves of these coffee plants.

## Materials and methods

### Plant material and growing conditions

D^T^ clone 14 and D^S^ clone 22 of *C. canephora* Conilon have been described previously (Marraccini *et al.*, 2011, 2012). For *Agrobacterium tumefaciens*-mediated transformation experiments, *C. arabica* var. Caturra plants were grown in *in vitro* conditions with a 12 h photoperiod (70 μmol m^−2^ s^−1^ light intensity) at 26 °C and 80% relative humidity (RH).

### DNA extraction and isolation of *CcDREB1D* promoter haplotypes

Leaves of D^T^ clone 14 and D^S^ clone 22 of *C. canephora* Conilon were used to extract genomic DNA and isolate *CcDREB1D* promoters. Genomic DNA was extracted from 20 mg of ground leaves as previously described by Cotta *et al.* (2014). For each coffee clone, *CcDREB1D* promoter haplotypes were amplified independently by PCR with high fidelity *Taq* Platinum® DNA polymerase according to the supplier’s instructions (Invitrogen) (initial denaturation: 94 °C–2 min followed by 30 cycles of 94 °C–30 s, 68 °C–30 s, 72 °C–3 min and a final extension step of 72 °C–10 min) using 100 ng of genomic DNA and 0.2 µM of the DREB-F1/DREB-R1 primers (Table 1). Amplified products were cloned into the pCR2.1-TOPO vector (Invitrogen) and propagated in the *E. coli* DH5α strain. As *C. canephora* is a diploid species, recombinant plasmids were extracted from 12 independent *E. coli* colonies in order to identify the two haplotypes of *CcDREB1D* promoter regions for each clone that were further double-strand sequenced using M13F and M13R universal primers and internal DREB-F2 and DREB-R2 primers (Table 1).

**Table 1.** List of primers used in this study

Primer	Sequence
DREB-F1^*a*^	5′ ACTCCTAGTAAGCGGCACGTTGTT 3′
DREB-R1^*a*^	5′ TGGCTTTGCAGGCATTGACTACG 3′
DREB-F2^*b*^	5′ TCGTGCATTCAACAGCACCGTCA 3′
DREB-R2^*b*^	5′ CCTTTCGTGGTTGTCTCTTGACCT 3′
S-DREB^*c*^	5′ TAATTCC*AAGCTT*TGTCTGAAGT 3′
M-DREB^*d*^	5′ AAGAGAACAAC*AAGCTT*CTTGT 3′
L-DREB^*e*^	5′ TCCTAGT*AAGCTT*CACGTTGT 3′
R-DREB^*f*^	5′ TGTTGAGAAATGGTT*AGATCT*TGAA 3′
GUS-F	5′ GCACTAGCGGGACTTTGCAA 3′
GUS-R	5′ CGCGAAGCGGGTAGATATCA 3′
DREBA09-F	5′ CAATGCCTGCAAAGCCAATTA 3′
DREBA09-R	5′ TTTTCCTGCCTGCACGTTTC 3′
GAPDH-F^*g*^	5′ TTGAAGGGCGGTGCAAA 3′
GAPDH-R^*g*^	5′ AACATGGGTGCATCCTTGCT 3′

^*a*^Primers used to amplify HP15, HP16 and HP17 haplotype promoters from the *CcDREB1D* (*Cc02_g03430*) gene of *C. canephora* (Denoeud *et al.*, 2014).

^*b*^Internal primers used for sequencing to verify the sequence of *CcDREB1D* promoters.

^*c*^Forward primer used to amplify the short (S: +182/−608) fragment of the *CcDREB1D* promoter haplotype. The *Hin*dII restriction site is italicized.

^*d*^Forward primer used to amplify the medium (M: +182/−966) fragment of the *CcDREB1D* promoter haplotype. The *Hin*dII restriction site is italicized.

^*e*^Forward primer used to amplify the long (L: +182/−1.308) fragment of the *CcDREB1D* promoter haplotype. The *Hin*dII restriction site is italicized.

^*f*^Reverse primer used to introduce the *Bgl*II restriction site. Other primer pairs were used in RT-qPCR experiments to determine gene expression levels of *uidA* (GUS-F/R) and *CcDREB1D* (DREBA09-F/R).

^*g*^The primer pair GAPDH-F/R was used to amplify the transcripts of the *CaGAPDH* (glyceraldehyde-3-phosphate dehydrogenase) gene used as reference to standardize the results of RT-qPCR experiments.

### Bioinformatic analysis

**Fig. 1.**
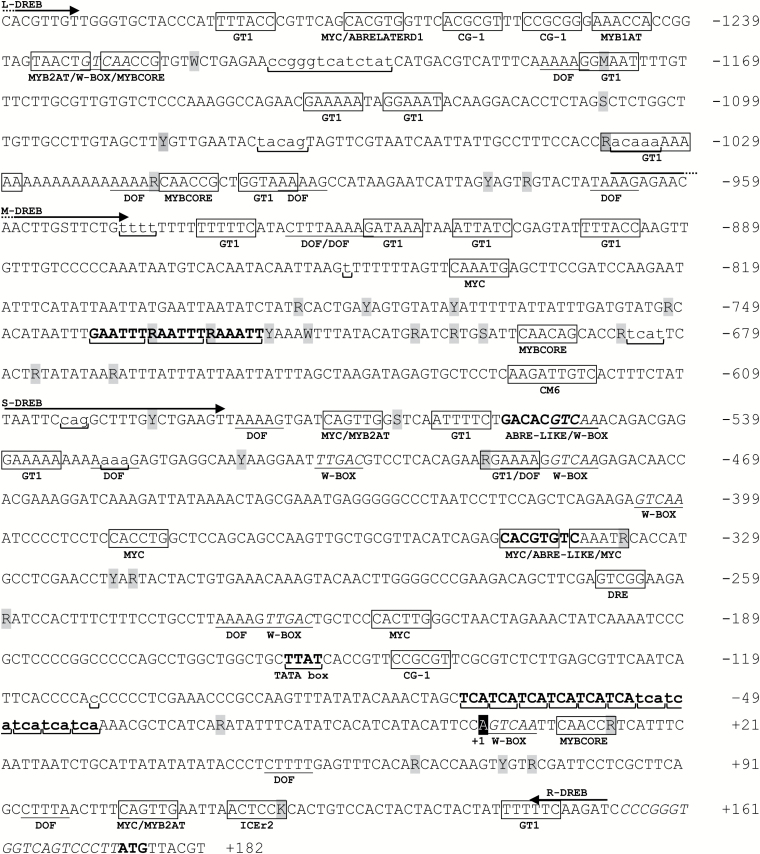
Consensus sequence of *CcDREB1D* promoter haplotypes. The consensus sequence contains all single nucleotide polymorphisms (SNPs, in gray) and insertion/deletions (INDELs, lower case underlined with horizontal brackets) found in the HP16 and HP17 haplotypes of *CcDREB1D* promoters. The nucleotides are numbered (on the right) in each lane using the first nucleotide (+1) of the *CcDREB1D* mRNA sequence (based on RNAseq data evidence available in the Coffee Genome Hub) as the start of numbering. CREs are identified by boxes with their corresponding name below except DOF/guard-cell (underlined), W-BOX (underlined and italics) and ABRE-LIKE (bold). Horizontal arrows indicate the primers (Table 1) used to amplify the full-length and truncated versions of coffee *CcDREB1D* promoter sequences (Fig. 2C). The TCA microsatellite GAAWTT unidentified motif and the putative TATA box are also indicated (bold and brackets). The 19 bp in italics upstream of the ATG (+174) of the β-glucuronidase corresponds to the multiple cloning site of the pBI121 vector.

The sequence of the unique gene *Cc02_g03430* (*CcDREB1D*: GSCOCP00020227001) from *C. canephora* (Denoeud *et al.*, 2014), available at Coffee Genome Hub (http://coffee-genome.org/), was analysed using the PlantPAN (http://plantpan.mbc.nctu.edu.tw, Chang *et al.*, 2008) and the PlantProm DB (http://www.softberry.com, Shahmuradov *et al.*, 2005) web interfaces. The DREB-F1/DREB-R1 primers (Table 1) were designed to amplify the region (−1308/+153 in Fig. 1) of *CcDREB1D* haplotypes from D^T^ clone 14 and D^S^ clone 22 that contained most of the *cis*-regulatory elements (CREs) supposed to be essential for their regulation by abiotic stress. The same web interfaces were used to predict the presence of CREs in the HP15, HP16 and HP17 haplotypes of the *CcDREB1D* promoters (Table 2). The significance of the putative CREs was evaluated through a maximum threshold of core similarity (equal to 1.0) and matrix similarity of 0.75.

**Table 2.** Cis*-regulatory elements (CREs) present in the* CcDREB1D *promoter haplotypes* The *cis*-regulatory elements present in the *CcDREB1D* promoter consensus sequence (Fig. 1) involved in osmotic- and cold-stress responsive gene expression were identified using PlantPAN (http://plantpan.mbc.nctu.edu.tw, Chang *et al.*, 2008) and the TSSP (http://www.softberry.com, Shahmuradov *et al.*, 2005) web interfaces. Experimental results supporting the involvement of CREs in the regulation of gene expression by abiotic stresses are also available in the literature (Ref.: 1, Sakuma *et al.*, 2002; 2, Yamaguchi-Shinozaki and Shinozaki, 2005; 3, Silva, 1994; 4, Plesch *et al.*, 2001; 5, Doherty *et al.*, 2009; 6, Higo *et al.*, 1999; 7, Chinnusamy *et al.*, 2007). Base abbreviations (IUPAC notation) are as follows: B=[not A], K=[G,T], M=[A,C], N=[any nucleotide], R=[A,G], V=[not T], W=[A,T], Y=[C,T]. TF: transcription factor. CREs were found either in sense (+) or antisense (−) DNA strands.

	CRE	Sequence	TF	Gene	Stress condition	Ref.	Sites	pHP15	pHP16	pHP17
ABRE	ABRELATERD1	ACGTG	bZIP	*erd1*	Water deficit, ABA	6	−1272/−1268 (+)	+	+	+
	ABRE-LIKE	BACGTGKM	bZIP	*rd29*	Water deficit, ABA	2	−557/−550 (−)	+	+	+
							−347/−340 (+)	+	+	+
DRE	DRE	CCGNC	ERF/AP2	*rd29A*	Water deficit, cold	1	−267/−263 (−)	+	+	+
	LTRECOREATCOR15	CCGAC	ERF/AP2	*cor15A*	Water deficit, cold	6				
MYB	MYBCORE	CNGTTR	bHLH	*rd22*	Water deficit, ABA	6	−1228/−1223 (−)	+	+	+
							−1012/−1007 (−)	+	+	+
							−695/−690 (−)	+	+	+
							+9/+14 (−)	−	−	+
	MYB1AT	WAACCA	bHLH (MYB15)	*rd22*	Water deficit, ABA	6	−1248/−1243 (+)	+	+	+
	MYB2AT	YAACTG	bHLH (MYB2)	*rd22*	Water deficit, ABA	6	−1235/−1230 (+)	+	+	+
							−575/−570 (−)	+	+	+
							+104/+109 (−)	+	+	+
MYC	MYCCONSENSUSAT	CANNTG	bHLH (ICE1)	*rd22*	Water deficit	6	−1273/−1268 (+)	+	+	+
							−842/−837 (+)	+	+	+
							−575/−570 (+)	+	+	+
							−387/−382 (+)	+	+	+
							−347/−342 (+)	+	+	+
							−340/−335 (+)	−	−	+
							−220/−215 (+)	+	+	+
							+104/+109 (+)	+	+	+
ICE	ICEr2	ACTCCG	bHLH (ICE)	*CBF2/ DREB1C*	Cold	7	+115/+120 (+)	+	+	−
CG-1	CG-1	VCGCGB	bHLH (CAMTA/SR1)	*PAL1*	CaM/Ca^2+^ inducible	3	−1263/−1258 (+)	+	+	+
							−1255/−1250 (+)	+	+	+
							−148/−143 (+)	+	+	+
CM6	CM6	AAGATTGTCA	unknown	unknown	Water deficit, ABA	5	−626/−617 (+)	+	+	+
GT-1	GT-1	GRWAAW	bHLH	*RBCS*	Light-inducible	6	−1286/−1281 (−)	+	+	+
							−1179/−1174 (+)	−	−	+
							−1137/−1132 (+)	+	+	+
							−1129/−1124 (+)	+	+	+
							−1037/−1027 (+)	+	+	−
							−1005/−999 (+)	+	+	+
							−938/−933 (−)	+	+	+
							−921/−916 (+)	+	+	+
							−912/−907 (−)	+	+	+
							−899/−894 (−)	+	+	+
							−564/−559 (−)	+	+	+
							−538/−533 (+)	+	+	+
							−489/−484 (+)	−	−	+
							+143/+148 (−)	+	+	+
DOF	DOF	WAAAG	ZF	*KST1*	Water deficit, ABA	4	−1183/−1179 (+)	+	+	+
							−1017/−1013 (+)	+	+	−
							−1000/−996 (+)	+	+	+
							−968/−964 (+)	+	+	+
							−929/−925 (−)	+	+	+
							−925/−921 (+)	+	+	+
							−584/−580 (+)	+	+	+
							−529/−525 (+)	+	+	+
							−487/−483 (+)	+	+	+
							−236/−232 (+)	+	+	+
							+49/+53 (−)	+	+	+
							+94/+98 (−)	+	+	+
W-BOX	W-BOX	TTGAC	ZF	*NPR1*	Wounding	6	−1230/−1226 (−)	+	+	+
							−552/−548 (−)	+	+	+
							−506/−502 (+)	+	+	+
							−482/−478 (−)	+	+	+
							−403/−399 (−)	+	+	+
							−231/−227 (+)	+	+	+
							+2/+6 (−)	+	+	+

### Construction of recombinant vectors

**Fig. 2.**
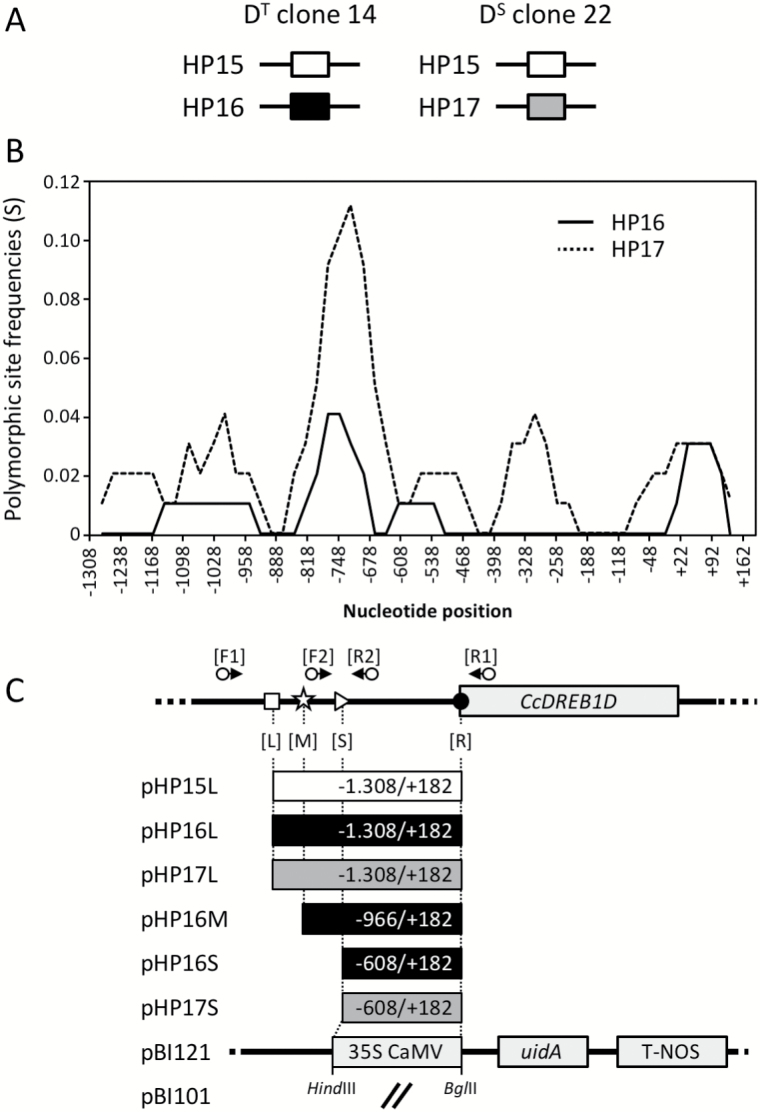
Haplotypes of *CcDREB1D* promoters of *C. canephora*. (A) *CcDREB1D* haplotypes found in drought-tolerant (D^T^) clone 14 (HP15/HP16) and drought-sensitive (D^S^) clone 22 (HP15/HP17) of *C. canephora*. Color code for haplotypes: HP15 (white), HP16 (black) and HP17 (gray). (B) Graphic representation of nucleotide variability detected in *CcDREB1D* promoter haplotypes. The *x*-axis corresponds to the bases of the HP15 sequence (Fig. 1). The *y*-axis corresponds to the frequency of polymorphic sites (S) observed in the HP16 (continuous line) and HP17 (dotted line) haplotypes compared with the HP15 sequence used as a reference. (C) Schematic representation of the *CcDREB1D* haplotypes analysed in transgenic plants of *C. arabica.* The schematic map of *CcDREB1D* (*Cc02_g03430*) is given in the upper part together with the DREB primers (F1, R1 in brackets, see Table 1) used to amplify the *CcDREB1D* promoter haplotypes. Plasmid names used for stable transformation of *C. arabica* are given for each construct, indicating the haplotype studied with its corresponding length (L, long; M, medium; S, short). The fragments were amplified using the forward primers (including the *Hin*dIII [AAGCTT] restriction site) corresponding to L-DREB (L, white square), M-DREB (M, white star) and S-DREB (S, white triangle) and the reverse primer R-DREB (including the *Bgl*II [AGATCT] restriction site) indicated by a black point and further cloned in front of the *uidA* reporter gene. pBI121 (CaMV35S:*uidA* gene construct) and pBI101 (*uidA* promoterless gene) were used as positive and negative controls of GUS enzymatic activities, respectively.

Binary vectors were generated by PCR using high fidelity *Taq* Platinum DNA polymerase amplification and recombinant plasmids harboring the HP15, HP16, and HP17 haplotypes of *CcDREB1D* promoters, as templates. The PCR reactions used the forward primers S-DREB, M-DREB, and L-DREB carrying the *Hin*dIII restriction site to amplify the short (S: +182/−608), medium (M: +182/−966), and long (L: +182/−1.308) fragments of the *CcDREB1D* promoter haplotypes, and the reverse primer R-DREB carrying the *Bgl*II restriction site (Table 1). The position of the R-DREB primer made it possible to include the entire 5′-UTR region of the *CcDREB1D* gene in the final constructs. PCR reactions were carried out using 10 ng of plasmids, *Taq* Platinum DNA polymerase, and primers as previously described, except that the first ten amplification cycles were carried out at an annealing temperature of 60 °C to ensure primer mismatch (calculated with the PrimerQuest^SM^® software, Integrated DNA Technologies). The resulting PCR products were subcloned into the pGEM-T Easy vector (Promega, Madison, WI, USA), digested with *Hin*dIII and *Bgl*II and cloned in the pBI121 vector (Clontech, Palo Alto, CA, USA) double-digested by the same enzymes. Following ligation and transformation in *E. coli*, the recombinant constructs were named according to their haplotype (HP) of the *CcDREB1D* promoter and length (S: short, M: medium, and L: long), namely as pHP16S and pHP17S (+182/−608, relative to ATG of the *uidA* reporter gene), pHP16M (+182/−966), and pHP15L, pHP16L and pHP17L (+182/−1308) (Fig. 2B). Each recombinant vector was then transferred independently by electroporation into competent cells of the disarmed *A. tumefaciens* strain LBA1119. After each cloning step, recombinant vectors were systematically extracted with the Wizard® Plus SV Minipreps DNA Purification System and *CcDREB1D* promoter fragments were verified by double-strand sequencing (Genome Express, France).

### *Agrobacterium tumefaciens*-mediated transformation and regeneration of *C. arabica*

*A. tumefaciens* strain LBA1119 harboring the recombinant vectors was grown at 28 °C for 24 h in yeast extract mannitol agar (YMB) selective medium with rifampicin (25 mg l^−1^) and kanamycin (50 mg l^−1^). Transgenic lines of *C. arabica* were generated by *A. tumefaciens*-mediated transformation using 12-month-old embryogenic calli competent for transformation as previously described (Etienne, 2005; Ribas *et al.*, 2011). At least eight independent putatively transformed cell lines were obtained for the six pHP constructs, as well as for coffee plants transformed by the pBI121 (*CaMV35S:uidA*) and pBI101 (*uidA*-promoterless) vectors used as positive and negative controls, respectively. Each kanamycin-resistant callus regenerated cotyledonary embryos giving plantlets after 4–5 months that were cultivated on MS medium with active charcoal (1 g l^−1^) under sterile conditions prior to testing for fast dehydration experiments. Once regenerated, DNA was extracted from leaf explants and successively tested by (i) PCR using the *nptII*- (kanamycin) and *uidA*-specific primers to confirm T-DNA integration in genomic DNA of *C. arabica*-transformed plantlets, and (ii) RT-qPCR to select transgenic lines harboring a single insertion of T-DNA (data not shown) representing around 50% of T1 transgenic coffee lines (Ribas *et al.* 2011).

### Abiotic assays used to study transformed plants of *C. arabica*

Regulation of the *CcDREB1D* promoter haplotypes was studied in primary transformants (T1) of *C. arabica* grown *in vitro* and subjected to the abiotic treatments described below. For each construct, 64 plantlets from eight independent transformation events were arranged in eight batches of eight plants. In each assay, each plant batch was subjected independently to water deficit for 0, 3, 6, 12, or 24 h, with 0 h (10.00 h, i.e. after 2 h of light exposure) corresponding to ‘no stress conditions’.

#### Fast dehydration

Genetically transformed *in vitro* plantlets with two to four pairs of leaves were placed in a laminar flow cabinet under a 0.49 m s^−1^ air flow for 1 h until reaching a visible dehydrated state. Each pHP construct was composed of five plantlets corresponding to independent transformation events.

#### Low RH assay (vapor pressure deficit)

A vapor pressure deficit (*D*) was induced by 9% RH at 27 °C under controlled conditions. A theoretical *D* was calculated applying the formula *D*=[1−(RH/100)]×*P*_WS_ (kPa), where *P*_WS_ (saturated vapor pressure)=3779 Pa at 28 °C. To create a 9% RH, 400 ml of KOH supersaturate solution was poured into the lower compartment of a 5 l bioreactor (Matis®, CID Plastiques, France) which remained sealed throughout the experiment. Plantlets were placed in the upper compartment of the bioreactor in 55 mm Petri dishes having their upper part exposed to the outside environment and radicles immersed in MS medium (Murashige and Skoog, 1962) through a small hole in the Petri dish cover. Seven plants were placed inside the bioreactor in the resulting 9% RH.

#### Polyethylene glycol assay

Osmotic potential was fixed at −2.0 MPa by adding polyethylene glycol (PEG) (relative molecular mass 6000; Sigma-Aldrich) to the MS medium, at 25 °C, using the empirical equations defined by Michel and Kaufmann (1973). Batches of seven plantlets were placed over 60 mg of vermiculite fully imbibed with 40 ml of PEG diluted in MS solution in sterile plastic Magenta® boxes.

### GUS staining

Histochemical GUS assays were performed with different tissues (root, stem, apical meristem, and leaf) derived from the same plant as those immersed in GUS staining solution (100 mM sodium phosphate buffer, pH 7.2, 10 mM sodium EDTA, 0.1% Triton X-100, 1 mg ml^−1^ 5-bromo-4-chloro-3-indolyl-D-glucuronic acid (Sigma-Aldrich), and 2.5 mM potassium ferrocyanidine). After infiltrating (vacuum for 2 × 10 min) the staining solution in plant tissues, the samples were incubated at 37 °C for 24 h, and then rinsed with water before image acquisition with a Retiga 2000R camera (G-Imaging Co., Wetzlar, Germany).

### Histology to reveal GUS in organs of transgenic coffee plants

Prior to observation, the GUS-stained samples were fixed (in 50% methanol and 10% acetic acid fixative) at 4 °C for 24 h, rinsed with water and then dehydrated (10 min in 50% ethanol, 10 min in 70% ethanol and 10 min in 90% ethanol). After observation with a Nikon binocular SMZ 1500 loupe, samples were embedded in 6% agarose for subsequent sections in a Microm HM650V vibrating blade microtome. For bright field microscopy observation, 50 µm-thick leaf sections were examined using a DM600 Leica microscope (Leica Microsystems GmbH, Wetzlar, Germany) and photographed. GUS expression patterns appeared highly conserved for the eight lines transformed with the same promoter construct.

### Proportion of GUS-stained guard cells in PEG and low RH assays

For bright field microscopy, GUS-stained leaves were fixed (50% methanol and 10% acetic acid) at 4 °C for 24 h, rinsed with water and incubated for at least 3 days in clearing solution (chloral hydrate:glycerol:water (4:1:2, v/v/v) to remove all leaf pigments. Prior to observation, tissues were rinsed with 70% ethanol and assembled on microscope slides for photography as described before. The proportion of GUS-stained guard cells on the abaxial epidermis of coffee leaves was calculated to estimate *CcDREB1D* promoter activity. For each pHP construct, the same three independent transgenic lines were studied along the entire kinetics. The proportion of GUS-stained guard cells (*p*) was obtained by *p*=*x*/*n*, where *x* is the number of stained guard cells and *n* the total number of guard cells (=150) per leaf zone. These values were assessed in 24 × 36 mm areas distributed in six pre-delimited leaf zones. For each pHP construct, three leaves from plants of three independent transformation events were sampled for each time (0, 3, 6, 12, and 24 h) in the PEG and low RH assays, and the respective proportion means (from 450 guard cells) and standard deviations for each time point were calculated.

### RNA isolation

Total RNA was extracted from leaves frozen in liquid nitrogen that were further ground and treated as described previously (Breitler *et al.*, 2016). RNA quantification was performed using a NanoDrop™ 1000 Spectrophotometer (Thermo Fisher Scientific, Waltham, MA, USA).

### Real-time RT-PCR assays

PCR experiments were performed as previously described by Marraccini *et al.* (2012). Primers (Table 1) were designed using Primer Express software (Applied Biosystems) to determine gene expression levels of *uidA* (GUS-F/R) and *CcDREB1D* (DREBA09-F/R). Data were analysed using SDS 2.1 software (Applied Biosystems) to determine cycle threshold (*C*_t_) values. The specificity of the PCR products generated for each set of primers was verified by analysing the *T*_m_ (dissociation) of amplified products. PCR efficiency (*E*) was estimated using absolute fluorescence data captured during the exponential phase of amplification of each reaction with the equation (1+*E*)=10^(−1/slope)^ (Ramakers *et al.*, 2003). Expression levels were calculated by applying the formula (1+*E*)^−ΔΔ*C*t^ where Δ*C*_t,target_=*C*_t,targetgene_−*C*_t,*CaGAPDH*_ and ΔΔ*C*_t_=Δ*C*_t,target_−Δ*C*_t,referencesample_, with the *T*_0_ samples being used as references for each construct. Expression levels were normalized with the expression of the *CaGAPDH* gene (GB accession number GW445811 using primer pair GAPDH-F/R) as the endogenous control (Cruz *et al.*, 2009).

## Results

### Comparison of DNA motifs and CREs between the different CcDREB1D promoter haplotypes isolated from drought-tolerant and drought-sensitive clones

The comparison of *CcDREB1D* promoter regions of *C. canephora* drought-tolerant (D^T^) clone 14 and drought-sensitive (D^S^) clone 22 revealed the existence of three haplotypes named HP15, HP16 and HP17, diverging from each other by several SNPs and INDELs (Fig. 1, and Supplementary Table S1 and Supplementary Fig. S1 at *JXB* online). The HP15 haplotype was common (without any SNPs) to both clones, while HP16 was specific to D^T^ clone 14 and HP17 specific to D^S^ clone 22 (Fig. 2A). Upstream of the first nucleotide (+1) of the *CcDREB1D* mRNA, a sliding window comparison of the haplotype sequences revealed two highly polymorphic domains (between base pairs −627/−806 and −932/−1121) concentrating most of the nucleotide variability (Fig. 2B), while the −115/−188 region was highly conserved between the three haplotypes, and the −188/−520 region was conserved between HP15 and HP16. Several CREs were also observed (Table 2) such as ABA-responsive (ABRE), dehydration-responsive (DRE), inducer of CBF expression region 2 (ICEr2), DNA-binding one zinc finger (DOF) binding sites (required for gene-specific expression in guard-cells), GT-1 binding sites (essential for light-inducible expression), the CG-1 element (also known as CAMTA: calmodulin binding transcription activator), and the conserved motif 6 (CM6), considered as a repressor of DREB1/CBF activation.

The comparison of *CcDREB1D* haplotypes revealed that the ICEr2 (+115/+120) and DOF (−1017/−1013) DNA boxes present in HP15 and HP16 were missing in HP17 (Table 2). However, HP17 displayed some elements, such as one MYBCORE (+9/+14) and one MYC (−340/−335) element, as well as two GT-1 binding sites (−489/−484 and −1179/−1174) not present in the HP15 and HP16 haplotypes (Table 2). Except for these differences, the HP15, HP16, and HP17 haplotypes had all CREs in common.

### Tissular activities of dehydration-induced *CcDREB1D* promoters in *C. arabica* transgenic plants

**Fig. 3.**
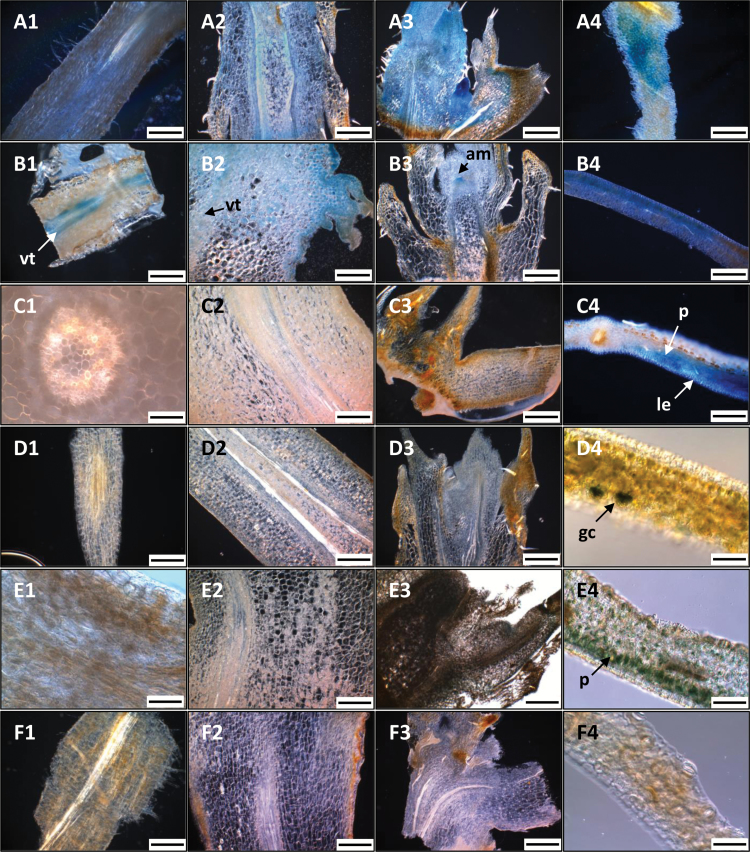
Histochemical localization of GUS activity in different tissues of transgenic *C. arabica* subjected to dehydration. Columns 1: root; 2: stem; 3: meristem; 4: leaf. The tissues belong to plants regenerated from constructs pHP17S (A), pHP16S (B), pHP16M (C), pHP16L (D), pHP15L (E), and pHP17L (F). The histochemical localization of GUS activity in control plants (WT, pBI101, and pBI121) is given in Supplementary Fig. S2. The scale bars given for each image correspond to 30 µm, excepted in (E4) and (D4) (80 µm), and (E3) (300 µm). Arrows indicate GUS staining restricted to specific cells and tissues: leaf epidermis (le), guard cell (gc), parenchyma (p), vascular tissue (vt), and apical meristem (am).

The activities of *CcDREB1D* promoter haplotypes were observed by comparing the GUS staining detected in *C. arabica* plants transformed by the short (pHP16S and pHP17S, Fig. 3A, B), the medium (pHP16M, Fig. 3C) and the full-length (pHP15L, pHP16L, and pHP17L, Fig. 3D–F) constructs and subjected to fast dehydration.

No staining was observed in dehydrated wild type plants and pBI101-transformed plants, whereas strong staining was seen in dehydrated plants transformed by pBI121 (Supplementary Fig. S2). In dehydrated pHP16S-transformed coffee plants, intense GUS staining was detected in root vascular tissues, while weak staining was observed in stem vascular tissues (Fig. 3B1, 2). In meristems, longitudinal-sections revealed weak GUS activity in leaf primordia and auxiliary buds, while intense staining was observed in apical meristems (Fig. 3B3). Strong GUS activities were also detected in leaf epidermis, as well as in spongy and palisade parenchyma (Fig. 3B4). GUS activities in pHP17S-transformed plants were similar (at spatial, tissue, and cell-specific levels) to those observed in pHP16S-transformed plants (Fig. 3A2, 4), except in roots where no staining was seen (Fig. 3A1).

**Fig. 4.**
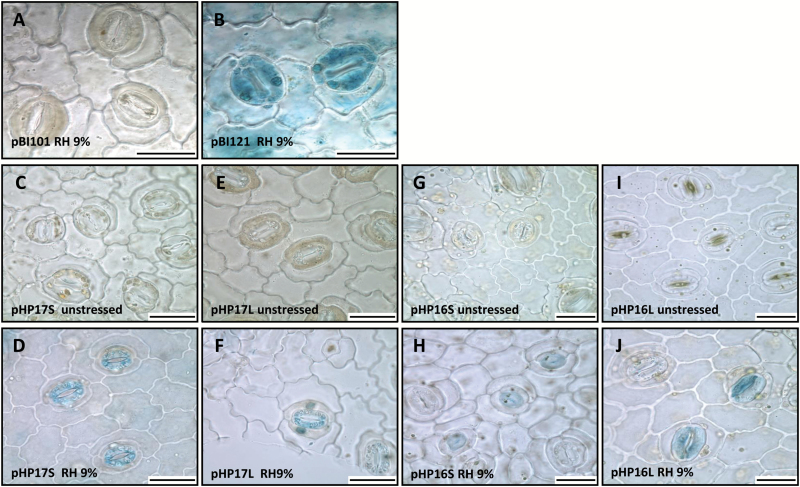
Histochemical localization of GUS activity in guard cells of *C. arabica* subjected to low air relative humidity (RH 9%). Guard cells visualized by bright field microscopy (×20 magnification) on the abaxial detached epidermis of coffee leaves. The explants were from coffee plants transformed by pBI101 (A, negative control), pBI121 (B, positive control), pHP17S (C, D), pHP17L (E, F), pHP16S (G, H), and pHP16L (I, J). Unstressed conditions for (C, E, G, I). Stressed conditions (9% RH) for (A, B, D, F, H, J). The scale bars in each image correspond to 30 µm.

For the pHP16L, pHP15L and pHP17L constructs, no GUS staining was observed in roots, stems, and meristems (Fig. 3D1–3, E1–3 and F1–3, respectively). Microscopic analyses of leaf transverse sections revealed GUS staining in palisade and spongy parenchyma of pHP15L-transformed plants (Fig. 3E4), and in several guard cells and stomata of pHP16L-transformed plants (Fig. 3D4).

The effects of promoter length were observed by comparing GUS activities in plants transformed by the pHP16S, pHP16M, and pHP16L constructs (Fig. 3B, C, D, respectively). For example, GUS activities were detected in leaves in pHP16M plants, particularly in the epidermis, in spongy and palisade parenchyma, and in the guard cells (Fig. 3C4) whereas no tissue specificity of staining was observed with pHP16S-transformed plants (Fig. 3B4). The greatest tissue specificity of GUS staining was shown for the plants transformed with the pHP16L construct, with histochemical staining observed in guard cells only (Fig. 3D4). The comparison of pHP17S- and pHP17L-transformed plants showed drastic reduction of GUS staining with the longer promoter (Fig. 3A and F, respectively).

### *CcDREB1D* promoters target *uidA* gene expression in guard cells of *C. arabica* under water deficit

In order to fine-tune the GUS staining results previously observed during the fast-drying assay, transformed coffee plants were subjected to controlled water deficit mimicked by low RH (9%) and further analysed to investigate GUS coloration in abaxial leaf surfaces. As controls, no staining was detected in plants transformed with the pBI101 vector (Fig. 4A) whereas strong staining was detected in guard and epidermis cells of leaves from pBI121-transformed plants (Fig. 4B). Under unstressed conditions, both epidermal cells and stomata were completely unstained in plants transformed by pHP17S, pHP17L, pHP16S, and pHP16L (Fig. 4C, E, G, I). After 24 h of reduced RH, GUS activities were clearly detected in guard cells of the same transgenic plants (Fig. 4D, F, H, J). GUS staining also appeared to be more intense in the colored stomata of pHP16L-transformed plants (Fig. 4J) than in those of pHP16S-transformed plants (Fig. 4H). For the three constructs, our results also clearly showed that in the same abaxial leaf area, stomata with stained guard cells coexisted with completely unstained stomata under a low RH, as observed for the HP16 haplotype (Fig. 4G, I). Longitudinal and cross sections of the pHP16L-transformed plants were also made to immunolocalize GUS proteins at the cellular level by using anti-GUS polyclonal antibody. This clearly detected GUS protein in guard cells with fluorescence signals that were highly concentrated in their thickened inner wall (Supplementary Fig. S3A). Fluorescence signals were not detected in all guard cells, but were observed in subsidiary cells, in spongy and palisade parenchyma cells, and in epidermis cells (Supplementary Fig. S3B).

### The activity of the HP16L *CcDREB1D* promoter was up-regulated by the low RH and PEG assays in leaves of transgenic *C. arabica*

**Fig. 5.**
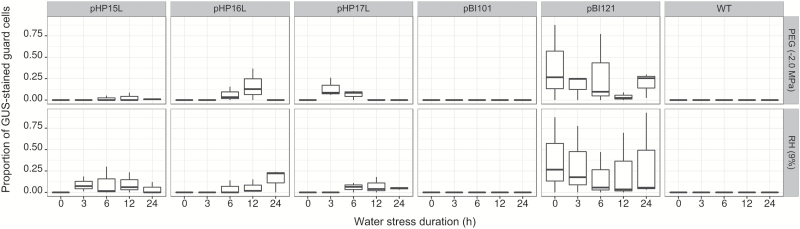
Proportions of GUS-stained guard cells: water deficit-induced regulation of GUS activity in mature leaf stomata driven by *CcDREB1D* promoter haplotypes. Change of GUS-stained guard cells’ proportions following 0, 3, 6, 12 and 24 h of exposure to PEG (equivalent to −2.0 MPa) and low air relative humidity (RH 9%) treatments in pHP15L-, pHP16L-, and pHP17L-transformed coffee plants. Box-and-whisker plots display variation in proportions of GUS-stained guard cells for each time assessed. The lower and upper hinges represent the first and third quartile respectively, the bold line represents the median, and the whiskers represent the smallest and the greatest values.

In order to see whether the nucleic acid polymorphisms existing in the three longest haplotypes (HP15L, HP16L, and HP17L) of the *CcDREB1D* promoter influenced the regulation of these sequences, the activity of these promoters was evaluated by calculating the proportion of GUS-stained guard cells on leaf abaxial regions in a time course response to water deficit assays simulated by PEG (equivalent to −2.0 MPa) and to low RH assays (Fig. 5). For the pHP16L- and pHP17L-transformed plants, the proportion of GUS-stained guard cells increased in the first hours of PEG treatment, reaching 20% and 10% maximum on average after 12 and 6 h, respectively (Fig. 5). However, the proportion of GUS-stained guard cells remained relatively low and stable during the PEG assay for pHP15L-transformed plants. For the low RH assay, around 20% of guard cells were GUS stained after 24 h of treatment in pHP16L-transformed plants. This percentage was reached after 6 h in pHP15L-transformed plants and gradually decreased over the time course to reach 5% after 24 h of stress. In pHP17L-transformed plants, the proportion of GUS-stained guard cells increased slowly to reach a mean of 10% after 12 h of low RH or after 5 h of PEG treatment and decreased thereafter.

### *uidA* and *CcDREB1D* gene expression

**Fig. 6.**
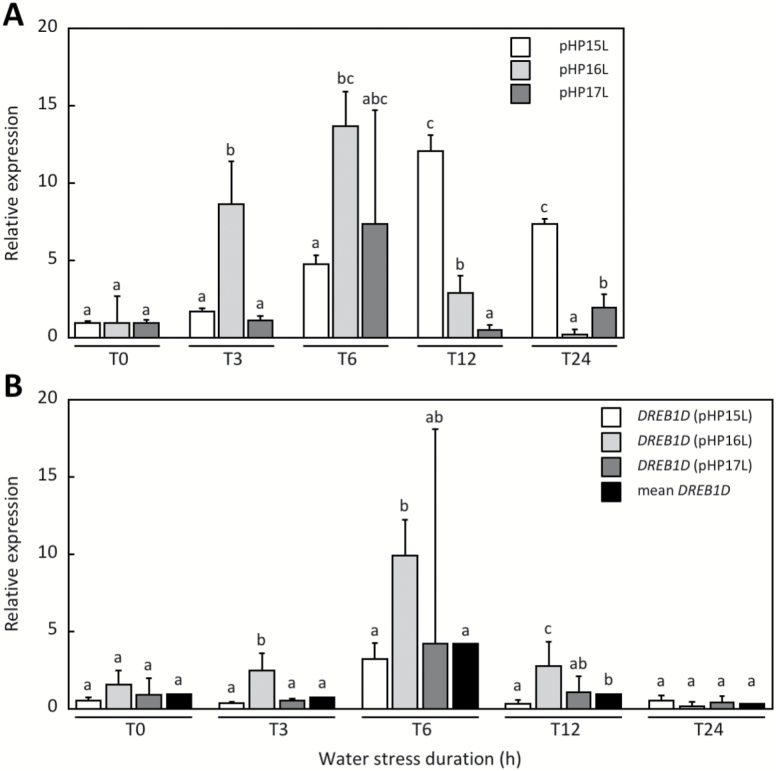
Expression profiles of the *uidA* and *CaDREB1D* genes in leaves of transgenic *C. arabica* during water deficit. Expression of the *uidA* (A) and *CaDREB1D* (B) genes was tested in leaves of coffee plants transformed by the pHP15L (white isobars), pHP16L (light gray isobars), and pHP17L (dark gray isobars) constructs and subjected to 0, 3, 6, 12, and 24 h osmotic stress (PEG treatment) by RT-qPCR experiments using the GUS-F/R and DREBA09-F/R primer pairs, respectively. Expression levels are indicated in relative quantification using the expression of the *CaGAPDH* gene as a reference. The results are expressed using *T*_0_ samples as internal calibrators for each construct. The relative quantification values correspond to the mean of at least three biological repetitions analysed by three technical replicates±SD. The significance of expression level differences was evaluated using the pairwise Wilcoxon rank test (non-parametric test). Treatments sharing the same letter are not significantly different.

The monitoring of *uidA* gene expression in leaves of coffee plants transformed by pHP15L, pHP16L, and pHP17L and subjected to PEG treatment showed low variability for each construct at each time of the water deficit bioassay. Similar expression profiles were obtained for all the constructs, showing a gradual increase in *uidA* gene expression with PEG treatment, which reached maximum expression after 6 h (pHP16L and pHP17L) or after 12 h (pHP15L) and declined thereafter (Fig. 6A). The main differences observed between the constructs concerned relative expression values in the first hours of stress (3 and 6 h), with *uidA* gene expression being higher in pHP16L-transformed plants than in pHP15L- and pHP17L-transformed plants. As controls, expression of the *CaDREB1D* endogenous gene increased with PEG treatment, reaching a peak at 6 h and then decreased gradually afterwards (Fig. 6B).

## Discussion

In our study, we investigated the responses of different haplotypes of the *CcDREB1D* (*CBF4*) promoters of *C. canephora* to PEG and low RH treatments by analysing their ability to regulate the expression of the *uidA* reporter gene in stably transformed *C. arabica* plants.

### Multiple CREs are involved in the regulation of *CcDREB1D* promoter haplotypes

The results presented here indicate that the HP15, HP16 and HP17 haplotypes of *CcDREB1D* promoters harbored several abiotic stress-responsive CREs in common, including those involved in tissue-specific and light regulation. They also contained ABRE and DRE elements, thereby suggesting possible regulation of these promoters by both ABA-dependent and -independent networks (Haake *et al.*, 2002; Yamaguchi-Shinozaki and Shinozaki, 2005). Interestingly, the co-occurrence in parallel strands and opposite direction of ABRE and DRE elements within 100 bp, identified herein for all haplotypes, is the most active structural organization observed for these CREs in drought-responsive genes (Mishra *et al.*, 2014). If we consider that DRE elements share a common DNA motif with low-temperature responsive element (LTRE), there could be a functional implication for coffee plant responses to cold stress, as observed for homologous genes in several plants (Zhang *et al.*, 2004; Ito *et al.*, 2006; Mao and Chen, 2012). This is reinforced by the fact that the three *CcDREB1D* haplotypes reported here also contain other CREs such as one ICEr2 box (except in the HP17 haplotype) and eight MYC-related binding sites (CANNTG), which are both motifs known to play a key role in the regulation of DREB1/CBF genes by cold-stress. Regarding MYC-related DNA motifs, two of them contained the preferable target sequence CAAATG (−842/−837 for all HPs and −340/−335 only for HP17) of the positive regulator inducer of CBF expression 1 (ICE1).

The three *CcDREB1D* haplotypes also contained several MYB-binding sites that can act as either an enhancer or repressor of promoter activity. One of them (MYB1AT: AAACCA) is a putative DNA binding site of the MYB15 *trans*-acting factor known to regulate negatively the expression of DREB1D/CBF genes (Agarwal *et al.*, 2006*a*). The remaining MYB boxes are potential positive regulators of DREB1/CBF expression, especially MYB2AT (YAACTG) binding sites required for activation of salt- and dehydration-responsive genes (Urao *et al.*, 1993; Abe *et al.*, 2003). *In silico* analyses of the *CcDREB1D* haplotypes also revealed the occurrence of several DOF-binding sites, already reported as key DNA motifs mediating guard cell-specific gene expression, and of several GT-1 binding sites essential for light-inducible expression, mainly located in the last 500 bp region (−921/−1183) of the *CcDREB1D* promoters (Plesch *et al.*, 2001; Galbiati *et al.*, 2008; Yang *et al.*, 2008; Gardner *et al.*, 2009; Cominelli *et al.*, 2011).

### Modular organization of DOF and light-induced DNA elements may account for preferential expression of *CcDREB1D* promoter haplotypes in guard cells

Previous studies showed that *OsDREB1B*, *AtDREB1C*, *AtDREB2C*, and *FeDREB1* promoters contained all CREs within the proximal regions (up to 1 kb) of these promoters necessary for enhancing gene expression by several abiotic stresses (Zarka *et al.*, 2003; Gutha and Reddy, 2008; Chen *et al.*, 2009; Chen *et al.*, 2012; Fang *et al.*, 2015). The results of GUS activities presented here in transgenic coffee plants transformed by the short (S: +182/−608), medium (M: +182/−966), or full-length (L: +182/−1308) sequences of *CcDREB1D* haplotypes clearly revealed the importance of the CREs located in the −608/−1308 region in regulating the expression of the *uidA* reporter gene in a tissue-specific manner. Indeed, while ubiquitous and strong GUS activities were observed in different tissues of coffee plants transformed by the shortest constructs (pHP16S and pHP17S), fine-tuned regulation of the *uidA* reporter gene was observed in coffee plants transformed by the full-length constructs (pHP15L, pHP16L, and pHP17L), with GUS activities restricted to leaf mesophyll and guard cells under stressed conditions. In that case, the degree of *CcDREB1D* haplotype activity in guard cells also appeared to increase with the number of DOF-binding sites, from the short (six sites) to the full-length (11/12 sites) promoter regions. One possible explanation could be an increase in the numbers of regulatory sites recognized by *trans*-acting factors known in the longest constructs of *CcDREB1D* haplotypes. For example, of the five MYB DNA boxes found within the −608/−1308 region, three MYBCORE and one MYB2AT are positive regulators and one (MYB1AT) is a putative binding site of MYB15 *trans*-acting factor known to repress *DREB1*/*CBF* genes (Agarwal *et al.*, 2006*a*) but also to regulate guard cell stomatal closure under drought conditions (Ding *et al.*, 2009). The presence of such DNA boxes could explain the low GUS activities observed in coffee plants transformed with the longest constructs (pHP15L, pHP16L, and pHP17L) compared with those observed in plants transformed with the shortest constructs (pHP16S and pHP17S). The increase in leaf-specific GUS activities observed from the short to the medium- and full-length constructs also tallied with the fact that cluster arrangements of DOF-binding sites are essential in determining guard cell-specific gene expression (Galbiati *et al.*, 2008; Yang *et al.*, 2008). However, this does not preclude the participation of other CREs functioning conjointly with DOF-binding sites to fine-tune the transcriptional regulation of gene expression in guard cells, as previously proposed in grape *VvMYB60*, potato *KST1* and cotton *GbSLSP* promoters, for example (Plesch *et al.*, 2001; Gardner *et al.*, 2009; Cominelli *et al.*, 2011; Galbiati *et al.*, 2011; Han *et al.*, 2013). Of the CREs that could play such roles, it is worth noting the presence of several GT-1 DNA binding sites in the *CcDREB1D* promoter haplotypes, with three of them (one in the medium (−608/−966) and two in the distal (−966/−1308) regions of the *CcDREB1D* promoters) closely located to DOF-binding elements. Therefore, GT-1 DNA binding sites could participate together with DOF elements in forming such clusters necessary for targeting guard cell-specific gene expression.

### The *CcDREB1D* promoter haplotypes respond differentially to RH and PEG abiotic stress

Few studies have reported the existence of functional variation between haplotypes (or alleles) of plant promoters in regulating gene expression differentially (de Meaux *et al.*, 2005; Takeshima *et al.*, 2016). The results presented here regarding the proportion of GUS-stained guard cells measured in transgenic coffee lines subjected to low water potential (PEG treatment) clearly showed greater activity for the HP16 and HP17 full-length haplotypes compared with very low activity for the HP15L haplotype. However, the activity of all the full-length *CcDREB1D* haplotypes was up-regulated under low air relative humidity (RH 9%). In that case, a slight increase in GUS-stained guard cells was observed in pHP15L- and pHP17L-transformed plants, while this proportion increased slightly but regularly in pHP16L-transformed plants in the RH treatment. The different time courses of GUS-stained guard cells observed between the HP15, HP16, and HP17 haplotypes during the PEG and RH treatments clearly revealed differences in the fine-tuning of the regulation of these *CcDREB1D* promoters, which were undoubtedly related to the nucleic acid polymorphism identified between these sequences directly affecting CREs and/or altering their cluster organization (see discussion below). Our results also indicated that the PEG and RH treatments affected the time course of activity of the HP16 and HP17 haplotypes of *CcDREB1D* promoters differently. For example, GUS-stained guard cells were detected earlier in pHP16L- and pHP17L-transformed plants in response to the PEG treatment compared with the RH treatment. PEG treatment is known to mediate low Ψ_w_ by reducing the osmotic potential of the nutrient solution, thereby mimicking low soil water potential that leads to a water deficit in the whole plant (Blum, 2014). Following the perception of water deficit by roots (major ABA producing organs), ABA would rapidly amplify the drought signal and activate the *CcDREB1D* promoter in guard cells, probably through the involvement of ABRE regulatory elements. The fact that exogenous ABA up-regulated the expression of the *uidA* and *CaDREB1D* gene, mainly in stomata guard cells of pHP16L-transformed coffee plants, supports the idea that this phytohormone plays a key role in coffee responses to water deficit (Torres *et al.*, 2016). While ABA induces early responses of *CcDREB1D* promoters by root-sensing water deficit, an inverse drought stimulus (where water stress is ‘sensed’ by aerial parts rather than roots) is expected to delay the response of the *CcDREB1D* promoter to water deficit. To simulate such conditions, our transgenic coffee plants were subjected to a water deficit of −2.0 MPa mimicked by the low RH treatment, which is known to increase the leaf-to-air water vapor pressure deficit (VPD), to generate evaporative demand and, consequently, to elevate the transpiration rate (Farooq *et al.*, 2009). The increase over time in GUS-stained guard cells in pHP16L- and pHP17L-transformed plants with the low RH (high VPD) treatment tends to support the existence of such a mechanism. However, while the frequency of GUS activity in guard cells increased gradually with a low RH in pHP16L-transformed plants, it increased in the first 12 h only and then decreased slowly in pHP17L-transformed plants, thereby demonstrating that these haplotypes differed in activity and/or regulation.

### The nucleic acid polymorphisms existing in *CcDREB1D* haplotypes are probably involved in modulating promoter activities

The comparison of HP15, HP16, and HP17 haplotype sequences revealed that (i) they had most of the essential CREs in common, and (ii) HP15 and HP16 were more closely related than HP17 regarding INDEL-type polymorphisms. By comparing CREs between HP16 and HP15, very few differences were observed regarding the boxes known to recognize key transcription factors involved in abiotic stress. The fact that these haplotypes of *CcDREB1D* promoters are regulated in a different manner in controlling the expression of the *uidA* reporter gene suggests that the nucleotide polymorphisms observed outside of the CREs might be responsible for these differences, as could be the case for the 2 bp insertion (AA: −1033/−1032) in HP16, with this INDEL being localized close to GT1 (−1037/−1027) and DOF (−1017/−1013) DNA binding sites. Several other polymorphisms, localized close to essential CREs, were also identified mainly in the polymorphic domains −627/−806 and −932/−1121. Within the −627/−806 region, the presence of (i) MYB- and MYC-like regulatory elements, respectively known as activator and repressors of transcription by drought, and (ii) a tandem (×3) repeat of the GAAWTT unidentified motif (−722/−739) only present in the HP16 haplotype is worth noting. Compared with the HP16 sequence, a G to A transition (in position −1013) in the HP17 haplotype, leading to the loss of one DOF domain, is also worth noting. Since DOF transcription factors are involved in the regulation of various plant processes (Le Hir and Bellini, 2013), such as stomatal functioning, for example (Gardner *et al.*, 2009; Negi *et al.*, 2013), this might explain the differences in activity observed between these two haplotypes under PEG and low RH assays.

### Drought stress up-regulates the expression of *CcDREB1D* haplotypes in transgenic plants of *C. arabica*

The increase in GUS activities observed in the water deficit mimicking treatments in leaves of transgenic coffee plants transformed by the pHP15L, pHP16L, and pHP17L constructs was confirmed by monitoring expression of the *uidA* reporter gene. For all the haplotypes, *uidA*-specific transcripts were accumulated after 3–12 h of PEG treatment (maximum after 6–12h) and declined thereafter. Although similar in terms of profiles, differences in promoter strength were found, with the HP16L promoter being stronger and occurring sooner (significant expression after 3 h) than the other two haplotypes. It is worth noting that the accumulation of *uidA* transcripts followed quite well the expression profile of the endogenous *CaDREB1D* gene, which was also up-regulated in the PEG treatment. In that case, the quantity of *CaDREB1D* transcripts detected after 24 h of treatment was very weak and did not differ from that before water deficit treatment. *CaDREB1D* transcripts were almost barely detectable after 6 h. Our results strongly suggest that the transient expression of the *CaDREB1D* gene under water deficit directly reflected the transitory activity of *DREB1D* promoters.

Even though the expression of *DREB1*/*CBF* genes has been commonly reported to be mainly induced by cold, our results clearly indicated that the expression of the *DREB1D* genes of *C. arabica* was rapidly induced by water deficit mimicking treatments and that the up-regulated expression of these genes was controlled at transcriptional level. These results also showed that the different promoter haplotypes of the *CcDREB1D* gene of the diploid *C. canephora* were correctly recognized by the transcriptional machinery of the allotetraploid species *C. arabica*.

Summarizing, the variations in intensities and staining patterns between the three haplotypes tested during this study suggested that HP16 (isolated from D^T^ clone 14 of *C. canephora*) had greater strength, as well as longer activity, under low RH conditions compared with HP15 (found in both D^T^ clone 14 and D^S^ clone 22) and HP17 (isolated from D^S^ clone 22) haplotypes. These characteristics might explain the higher stomatal conductance (*g*_s_), and consequently the more efficient mechanisms in controlling the transpiration rate observed under water deficit in D^T^ clone 14 compared with D^S^ clone 22 (Marraccini *et al.*, 2012). Based on the results of this study, work is now on-going to analyse the genetic diversity of *DREB1D* promoters in other D^T^ and D^S^ plants of *Coffea* species with the objective to identify molecular markers for breeding drought-resistant cultivars, and also to study the effects of other types of abiotic stress (e.g. high temperature and light intensities, cold stress, etc.) on the regulation and activity of the *CcDREB1D* haplotypes.

## Supplementary data

Supplementary data are available at *JXB* online.

Fig. S1. Nucleic acid alignments of *CcDREB1D* haplotypes (HP15, HP16 and HP17) with the *CcDREB1D* consensus sequence (see Fig. 1).

Fig. S2. Histochemical localization (root, stem, meristem, leaf) of GUS activity in dehydrated control plants of *C. arabica*.

Fig. S3. Confocal laser scanning microscopy (CLSM) micrographs from leaf cross-sections with immunolocalization of GUS protein.

Table S1. List of nucleic acid polymorphisms found in the HP15, HP16, and HP17 haplotypes of *CcDREB1D* coffee promoters.

## Accession numbers

Sequence data for the HP15, HP16, and HP17 haplotypes of *CcDREB1D* promoters can be found in the GenBank database under accession numbers KM281308, KM281309, and KM281311, respectively.

## Author contributions

GSCA cloned and sequenced the *CcDREB1D* promoter haplotypes, made the constructs and analysed these sequences. GSCA, LFT, HE, and ED carried out the genetic transformation of *C. arabica*, applied abiotic stress, and carried out GUS staining and microscopy analyses with the help of FG. LFT extracted RNA samples used by J-CB to perform qPCR experiments and analysed the results together with TJ. HE, BB, ACA and PM designed the study, drew up the experimental design and implemented it. GSCA, ACA, TJ, PM, and HE wrote the article, which was approved by all the authors.

## Supplementary Material

Supplementary_Table_S1_Figures_S1_S3

## Abbreviations:

ABA: abscisic acid
ABRE: ABA-responsive element
CBF: cold-binding factor
CRE: *cis*-regulatory element
DOF: DNA-binding one zinc finger
DRE: dehydration-responsive element
DREB: dehydration responsive element binding transcription factor
GUS: β-glucuronidase
ICE: inducer of CBF expression
PEG: polyethylene glycol
RH: relative humidity
SNP: single-nucleotide polymorphism
VPD: vapor pressure deficit.
