# Supplementary material for: Differential fine-tuning of gene expression regulation in coffee leaves by CcDREB1D promoter haplotypes under water deficit
Source: J Exp Bot. 2017 Jul 11;68(11):3017–31. doi: 10.1093/jxb/erx166 (PMC5853422; doi:10.1093/jxb/erx166)

Differential fine tune regulation of gene expression in coffee leaves by CcDREB1D promoter haplotypes under drought

Gabriel Sergio Costa Alves, Luana Ferreira Torres, Eveline Déchamp, Jean-Christophe Breitler, Thierry Joët, Frédéric Gatineau, Alan Carvalho Andrade, Benoît Bertrand, Pierre Marraccini, and Hervé Etienne

Supplementary Data

Supplementary Table S1. List of nucleic polymorphisms found in the HP15, HP16, and HP17 haplotypes of CcDREB1D coffee promoters.

Supplementary Table S1. List of nucleic polymorphisms found in the HP15, HP16, and HP17 haplotypes of *CcDREB1D* coffee promoters.

Polymorphism	Consensus Sequence	Variant (s)	Polymorphism Type	Minimum	Maximum	Length	HP15	HP16	HP17
A -> T	W	T	SNP (transversion)	-1,219	-1,219	1	A	A	T
-CCGGGTCATCTAT	CCGGGTCATCTAT	-------------	Deletion	-1,211	-1,199	13	CCGGGTCATCTAT	CCGGGTCATCTAT	-------------
C -> A	M	A	SNP (transversion)	-1,177	-1,177	1	C	C	A
C -> G	S	G	SNP (transversion)	-1,107	-1,107	1	C	C	G
T -> C	Y	C	SNP (transition)	-1,082	-1,082	1	T	C	T
-TACAG	TACAG	-----	Deletion	-1,072	-1,068	5	TACAG	TACAG	-----
-ACAAA	---AA	ACAAA	Insertion	-1,036	-1,032	5	-----	---AA	ACAAA
G -> A	R	A	SNP (transition)	-1,013	-1,013	1	G	G	A
T -> C	Y	C	SNP (transition)	-984	-984	1	T	C	T
A -> G	R	G	SNP (transition)	-980	-980	1	A	A	G
C -> G	S	G	SNP (transversion)	-957	-957	1	C	C	G
(TT)4 -> (TT)6	----	TTTT	Insertion (tandem repeat)	-946	-943	4	----	----	TTTT
(T)6 -> (T)7	-	T	Insertion (tandem repeat)	-853	-853	1	-	T	T
A -> G	R	G	SNP (transition)	-788	-788	1	A	A	G
T -> C	Y	C	SNP (transition)	-781	-781	1	T	T	C
C -> T	Y	T	SNP (transition)	-772	-772	1	C	T	T
G -> A	R	A	SNP (transition)	-750	-750	1	G	G	A
A -> G	R	G	SNP (transition)	-733	-733	1	A	G	G
G -> A	R	A	SNP (transition)	-727	-727	1	G	G	A
C -> T	Y	T	SNP (transition)	-721	-721	1	C	C	T
A -> T	W	T	SNP (transversion)	-717	-717	1	A	T	T
G -> A	R	A	SNP (transition)	-706	-706	1	G	A	A
G -> A	R	A	SNP (transition)	-702	-702	1	G	G	A
C -> G	S	G	SNP (transversion)	-699	-699	1	C	C	G

Supplementary Table S1 (continued)

Polymorphism	Consensus Sequence	Variant (s)	Polymorphism Type	Minimum	Maximum	Length	HP15	HP16	HP17
G -> ATCAT	R----	ATCAT	Insertion	-685	-681	5	G----	G----	ATCAT
A -> G	R	G	SNP (transition)	-675	-675	1	A	A	G
A -> G	R	G	SNP (transition)	-667	-667	1	A	A	G
-CAG	CAG	---	Deletion	-602	-600	3	CAG	---	CAG
T -> C	Y	C	SNP (transition)	-593	-593	1	T	T	C
G -> C	S	C	SNP (transversion)	-568	-568	1	G	C	G
(AAA)3 -> (AAA)4	---	AAA	Insertion (tandem repeat)	-528	-526	3	---	---	AAA
T -> C	Y	C	SNP (transition)	-514	-514	1	T	T	C
A -> G	R	G	SNP (transition)	-489	-489	1	A	A	G
A -> G	R	G	SNP (transition)	-335	-335	1	A	A	G
T -> C	Y	C	SNP (transition)	-317	-317	1	T	T	C
A -> G	R	G	SNP (transition)	-315	-315	1	A	A	G
A -> G	R	G	SNP (transition)	-258	-258	1	A	A	G
(C)5 -> (C)6	-	C	Insertion (tandem repeat)	-109	-110	1	-	C	C
(TCA)6 -> (TCA)11	---------------	ATCATCATCATCATC	Insertion (tandem repeat)	-53	-39	15	---------------	---------------	ATCATCATCATCATC
A -> G	R	G	SNP (transition)	-26	-26	1	A	A	G
A -> G	R	G	SNP (transition)	+14	+14	1	A	A	G
G -> A	R	A	SNP (transition)	+64	+64	1	G	A	A
C -> T	Y	T	SNP (transition)	+73	+73	1	C	T	C
G -> A	R	A	SNP (transition)	+76	+76	1	G	A	A
G -> T	K	T	SNP (transversion)	+120	+120	1	G	G	T

Supplementary Fig. S1. Nucleic alignments of CcDREB1D haplotypes (HP15, HP16 and HP17) with the CcDREB1D consensus sequence (see Fig. 1).

Supplementary Fig. S2. Histochemical localization (root, stem, meristem, leaf) of GUS activity in dehydrated control plants of C. arabica. No staining in untransformed plants (A: wild type) and in pB101-transformed plants (B) while strong staining was observed in all tissues of pB121-transformed coffee plants. The scale bars given for each image correspond to 30 µm.

Supplementary Fig. S3. Confocal laser scanning microscopy (CLSM) micrographs from leaf cross-sections with immunolocalization of GUS protein. Leaves of pHP16L-transformed plants were sampled after 24 h of exposure to 9% RH. Fluorescence immunolabeling of GUS proteins was carried out with 5% bovine serum albumin (BSA) in PBS (blocking buffer, 3 h), anti-β-glucuronidase rabbit antibody (1:200 in blocking buffer, overnight at 4°C), PBS (3 × 15 min washing). Leaves were also treated with anti-GUS rabbit antibody and then with a secondary anti-rabbit IgGs mouse antibody coupled to an Alexa Fluor® 488 probe (4 µg.ml−1 in 2% BSA in PBS, 1 h in the dark), and PBS (3 × 15 min washing). Sections were mounted in PBS and observed under an LSM510 META NLO Axiovert 200M Inverted Microscope (Carl Zeiss, Germany), equipped with a Chameleon Ultra II laser (Coherent, Glasgow, UK) with the following settings: laser 488 nm, BP 500-530 nm. Micrograph deconvolution of spectrum signals was achieved using the Linear Unmixing technique (A and B). Cells testing positive for GUS green fluorescence are indicated by white arrows. Epidermal cell (ec); guard cell (gc); parenchyma cell (pc).

Supplementary Figure S1

```
Consensus  .         .         .         .         .         .         .              -1239
pHP17L     CACGTTGTTGGGTGCTACCCATTTTACCCGTTCAGCACGTGGTTCACGCGTTTCCGCGGGAAACCACCGG     -1239
pHP15L     CACGTTGTTGGGTGCTACCCATTTTACCCGTTCAGCACGTGGTTCACGCGTTTCCGCGGGAAACCACCGG     -1239
pHP16L     CACGTTGTTGGGTGCTACCCATTTTACCCGTTCAGCACGTGGTTCACGCGTTTCCGCGGGAAACCACCGG     -1239

Consensus  .         .        W.       cgggtcatctatc         .         .M             -1169
pHP17L     TAGTAACTGTCAACCGTGTTCTGAGAAC-------------ATGACGTCATTTCAAAAAGGAAATTTTGT     -1182
pHP15L     TAGTAACTGTCAACCGTGTACTGAGAACCGGGTCATCTATCATGACGTCATTTCAAAAAGGCAATTTTGT     -1169
pHP16L     TAGTAACTGTCAACCGTGTACTGAGAACCGGGTCATCTATCATGACGTCATTTCAAAAAGGCAATTTTGT     -1169

Consensus  .         .         .         .         .         .         .S             -1099
pHP17L     TTCTTGCGTTGTGTCTCCCAAAGGCCAGAACGAAAAATAGGAAATACAAGGACACCTCTAGGCTCTGGCT     -1112
pHP15L     TTCTTGCGTTGTGTCTCCCAAAGGCCAGAACGAAAAATAGGAAATACAAGGACACCTCTAGCCTCTGGCT     -1099
pHP16L     TTCTTGCGTTGTGTCTCCCAAAGGCCAGAACGAAAAATAGGAAATACAAGGACACCTCTAGCCTCTGGCT     -1099

Consensus  .         .     Y   .      acagt        .         .         .Racaaa        -1029
pHP17L     TGTTGCCTTGTAGCTTTGTTGAATACT-----AGTTCGTAATCAATTATTGCCTTTCCACCAACAAAAAA     -1047
pHP15L     TGTTGCCTTGTAGCTTTGTTGAATACTACAGTAGTTCGTAATCAATTATTGCCTTTCCACCG-----AAA     -1034
pHP16L     TGTTGCCTTGTAGCTTCGTTGAATACTACAGTAGTTCGTAATCAATTATTGCCTTTCCACCG---AAAAA     -1032

Consensus  .         .    R    .         .         .        Y.  R      .               -959
pHP17L     AAAAAAAAAAAAAAAACAACCGCTGGTAAAAAGCCATAAGAATCATTAGTAGTGGTACTATAAAGAGAAC      -977
pHP15L     AAAAAAAAAAAAAAAAGCAACCGCTGGTAAAAAGCCATAAGAATCATTAGTAGTAGTACTATAAAGAGAAC      -964
pHP16L     AAAAAAAAAAAAAAAAGCAACCGCTGGTAAAAAGCCATAAGAATCATTAGCAGTAGTACTATAAAGAGAAC      -962

Consensus  .     S   . tttt    .         .         .         .         .               -889
pHP17L     AACTTGGTTCTGTTTTTTTTTTTTTTCATACTTTAAAAGATAAATAAATTATCCGAGTATTTTACCAAGTT      -907
pHP15L     AACTTGCTTCTG----TTTTTTTTTTCATACTTTAAAAGATAAATAAATTATCCGAGTATTTTACCAAGTT      -898
pHP16L     AACTTGCTTCTG----TTTTTTTTTTCATACTTTAAAAGATAAATAAATTATCCGAGTATTTTACCAAGTT      -896

Consensus  .         .         .         .    t    .         .         .               -819
pHP17L     GTTTGTCCCCCAAATAATGTCACAATACAATTAAGTTTTTTTAGTTCAAATGAGCTTCCGATCCAAGAAT      -837
pHP15L     GTTTGTCCCCCAAATAATGTCACAATACAATTAAG-TTTTTTAGTTCAAATGAGCTTCCGATCCAAGAAT      -829
pHP16L     GTTTGTCCCCCAAATAATGTCACAATACAATTAAGTTTTTTTAGTTCAAATGAGCTTCCGATCCAAGAAT      -826

Consensus  .         .         .         R      Y  .     Y   .         .       R       -749
pHP17L     ATTTCATATTAATTATGAATTAATATCTATGCACTGACAGTGTATATATTTTTATTATTTGATGTATGAC      -767
pHP15L     ATTTCATATTAATTATGAATTAATATCTATACACTGATAGTGTATACATTTTTATTATTTGATGTATGGC      -759
pHP16L     ATTTCATATTAATTATGAATTAATATCTATACACTGATAGTGTATATATTTTTATTATTTGATGTATGGC      -756
```

Supplementary Figure 1 (continued)

```
Consensus      .              R    .R     Y  .W        . R   R  S.         .  Rtcat          -679
pHP17L         ACATAATTTGAATTTGAATTTAAAATTTAAATTTTATACATGAATCATGGATTCAACAGCACCATCATTC        -697
pHP15L         ACATAATTTGAATTTAAATTTGAAATTCAAAATTTATACATGGATCGTGCATTCAACAGCACCG----TC        -693
pHP16L         ACATAATTTGAATTTGAATTTGAAATTCAAATTTTATACATGAATCGTGCATTCAACAGCACCG----TC        -690

Consensus      .  R      .R        .         .         .         .         .                 -609
pHP17L         ACTGTATATAAGATTTATTTATTAATTATTTAGCTAAGATAGAGTGCTCCTCAAGATTGTCACTTTCTAT        -627
pHP15L         ACTATATATAAAATTTATTTATTAATTATTTAGCTAAGATAGAGTGCTCCTCAAGATTGTCACTTTCTAT        -623
pHP16L         ACTATATATAAAATTTATTTATTAATTATTTAGCTAAGATAGAGTGCTCCTCAAGATTGTCACTTTCTAT        -620

Consensus      .     cag .    Y    .         .         S         .         .                 -539
pHP17L         TAATTCCAGGCTTTGCCTGAAGTTAAAAGTGATCAGTTGGGTCAATTTTCTGACACGTCAAACAGACGAG        -557
pHP15L         TAATTCCAGGCTTTGTCTGAAGTTAAAAGTGATCAGTTGGGTCAATTTTCTGACACGTCAAACAGACGAG        -553
pHP16L         TAATTC---GCTTTGTCTGAAGTTAAAAGTGATCAGTTGGCTCAATTTTCTGACACGTCAAACAGACGAG        -553

Consensus      .aaa      .         .   Y     .         .        R.         .                 -469
pHP17L         GAAAAAAAAAAAAGAGTGAGGCAACAAGGAATTTGACGTCCTCACAGAAGGAAAAGGTCAAGAGACAACC        -487
pHP15L         G---AAAAAAAAAGAGTGAGGCAATAAGGAATTTGACGTCCTCACAGAAAGAAAAGGTCAAGAGACAACC        -486
pHP16L         G---AAAAAAAAAGAGTGAGGCAATAAGGAATTTGACGTCCTCACAGAAAGAAAAGGTCAAGAGACAACC        -486

Consensus      .         .         .         .         .         .         .                 -399
pHP17L         ACGAAAGGATCAAAGATTATAAAACTAGCGAAATGAGGGGGCCCTAATCCTTCCAGCTCAGAAGAGTCAA        -417
pHP15L         ACGAAAGGATCAAAGATTATAAAACTAGCGAAATGAGGGGGCCCTAATCCTTCCAGCTCAGAAGAGTCAA        -416
pHP16L         ACGAAAGGATCAAAGATTATAAAACTAGCGAAATGAGGGGGCCCTAATCCTTCCAGCTCAGAAGAGTCAA        -416

Consensus      .         .         .         .         .         .         .  R              -329
pHP17L         ATCCCCTCCTCCACCTGGCTCCAGCAGCCAAGTTGCTGCGTTACATCAGAGCACGTGTCAAATGCACCAT        -347
pHP15L         ATCCCCTCCTCCACCTGGCTCCAGCAGCCAAGTTGCTGCGTTACATCAGAGCACGTGTCAAATACACCAT        -346
pHP16L         ATCCCCTCCTCCACCTGGCTCCAGCAGCCAAGTTGCTGCGTTACATCAGAGCACGTGTCAAATACACCAT        -346

Consensus      .         .Y R      .         .         .         .         .                 -259
pHP17L         GCCTCGAACCTCAGTACTACTGTGAAACAAAGTACAACTTGGGGCCCGAAGACAGCTTCGAGTCGGAAGA        -277
pHP15L         GCCTCGAACCTTAATACTACTGTGAAACAAAGTACAACTTGGGGCCCGAAGACAGCTTCGAGTCGGAAGA        -276
pHP16L         GCCTCGAACCTTAATACTACTGTGAAACAAAGTACAACTTGGGGCCCGAAGACAGCTTCGAGTCGGAAGA        -276

Consensus      R         .         .         .         .         .         .                 -189
pHP17L         GATCCACTTTCTTTCCTGCCTTAAAAGTTGACTGCTCCCACTTGGGCTAACTAGAAACTATCAAAATCCC        -207
pHP15L         AATCCACTTTCTTTCCTGCCTTAAAAGTTGACTGCTCCCACTTGGGCTAACTAGAAACTATCAAAATCCC        -206
pHP16L         AATCCACTTTCTTTCCTGCCTTAAAAGTTGACTGCTCCCACTTGGGCTAACTAGAAACTATCAAAATCCC        -206
```

Supplementary Figure 1 (continued)

```
Consensus  .         .         .         .         .         .         .               -119
pHP17L     GCTCCCCGGCCCCCAGCCTGGCTGGCTGCTTATCACCGTTCCGCGTTCGCGTCTCTTGAGCGTTCAATCA          -137
pHP15L     GCTCCCCGGCCCCCAGCCTGGCTGGCTGCTTATCACCGTTCCGCGTTCGCGTCTCTTGAGCGTTCAATCA          -136
pHP16L     GCTCCCCGGCCCCCAGCCTGGCTGGCTGCTTATCACCGTTCCGCGTTCGCGTCTCTTGAGCGTTCAATCA          -136

Consensus  .        C.         .         .         .         .         .   atcatc           -49
pHP17L     TTCACCCCACCCCCCTCGAAACCCGCCAAGTTTATATACAAACTAGCTCATCATCATCATCATCATCATC           -67
pHP15L     TTCACCCCA-CCCCCTCGAAACCCGCCAAGTTTATATACAAACTAGCTCATCATCATCATCATC------           -73
pHP16L     TTCACCCCACCCCCCTCGAAACCCGCCAAGTTTATATACAAACTAGCTCATCATCATCATCATC------           -72

Consensus  atcatcatc .         . R       .         .         .         . R                 +21
pHP17L     ATCATCATCAAAACGCTCATCAGATATTTCATATCACATCATACATTCCAGTCAATTCAACCGTCATTTC            +3
pHP15L     ---------AAAACGCTCATCAAATATTTCATATCACATCATACATTCCAGTCAATTCAACCATCATTTC           -12
pHP16L     ---------AAAACGCTCATCAAATATTTCATATCACATCATACATTCCAGTCAATTCAACCATCATTTC           -11

Consensus  .         .         .         .         . R       .Y  R     .                   +91
pHP17L     AATTAATCTGCATTATATATATACCCTCTTTTGAGTTTCACAACACCAAGTCGTACGATTCCTCGCTTCA           +73
pHP15L     AATTAATCTGCATTATATATATACCCTCTTTTGAGTTTCACAGCACCAAGTCGTGCGATTCCTCGCTTCA           +58
pHP16L     AATTAATCTGCATTATATATATACCCTCTTTTGAGTTTCACAACACCAAGTTGTACGATTCCTCGCTTCA           +59

Consensus  .         .         .       K .         .         .         .                  +161
pHP17L     GCCTTTAACTTTCAGTTGAATTAACTCCTCACTGTCCACTACTACTACTATTTTTTCAAGATCCCCGGGT          +143
pHP15L     GCCTTTAACTTTCAGTTGAATTAACTCCGCACTGTCCACTACTACTACTATTTTTTCAAGATCCCCGGGT          +128
pHP16L     GCCTTTAACTTTCAGTTGAATTAACTCCGCACTGTCCACTACTACTACTATTTTTTCAAGATCCCCGGGT          +129

Consensus  .         .         .       +182
pHP17L     GGTCAGTCCCTTATGTTACGT       +164
pHP15L     GGTCAGTCCCTTATGTTACGT       +149
pHP16L     GGTCAGTCCCTTATGTTACGT       +150
```

Suppl. Fig. S2

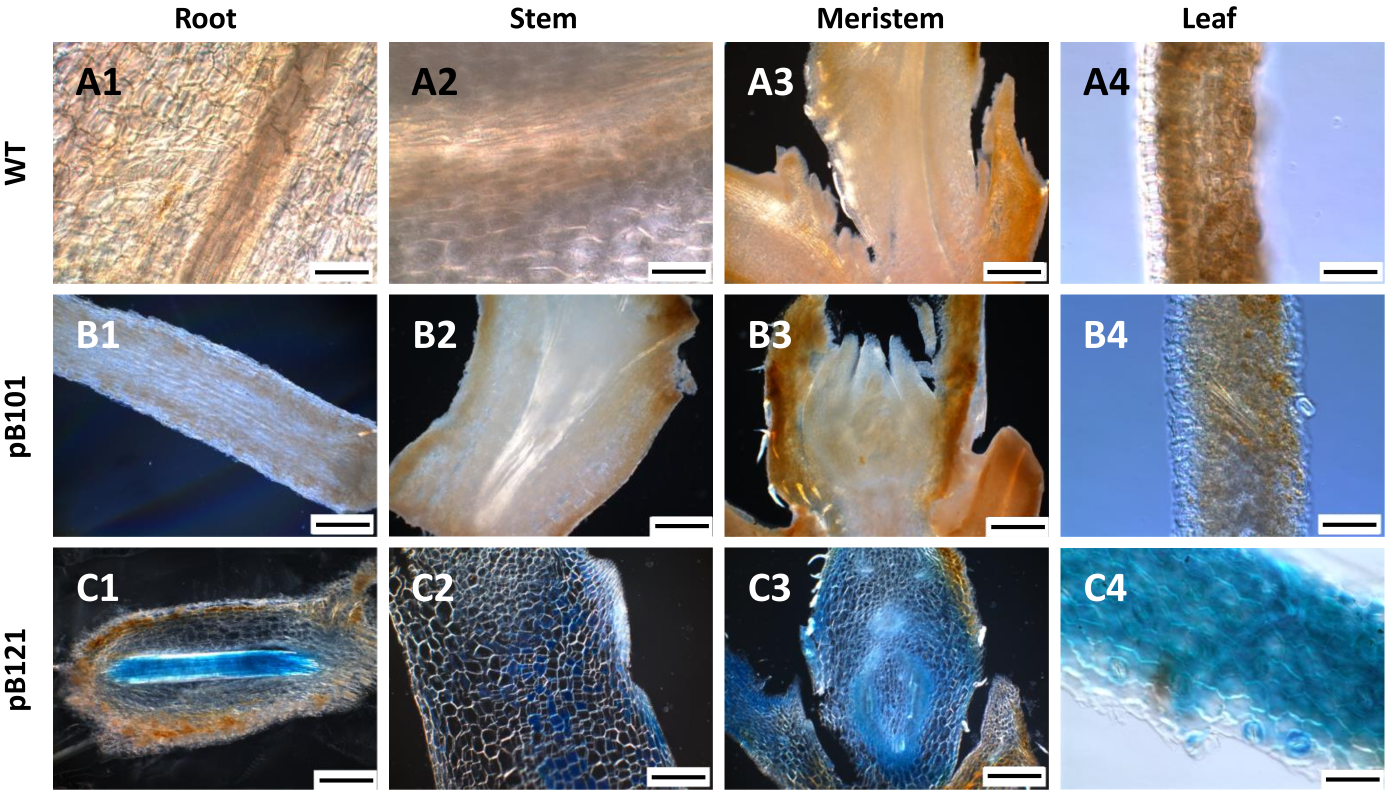
Root
Stem
Meristem
Leaf
WT
pB101
pB121
A1
A2
A3
A4
B1
B2
B3
B4
C1
C2
C3
C4

Suppl. Fig. 3

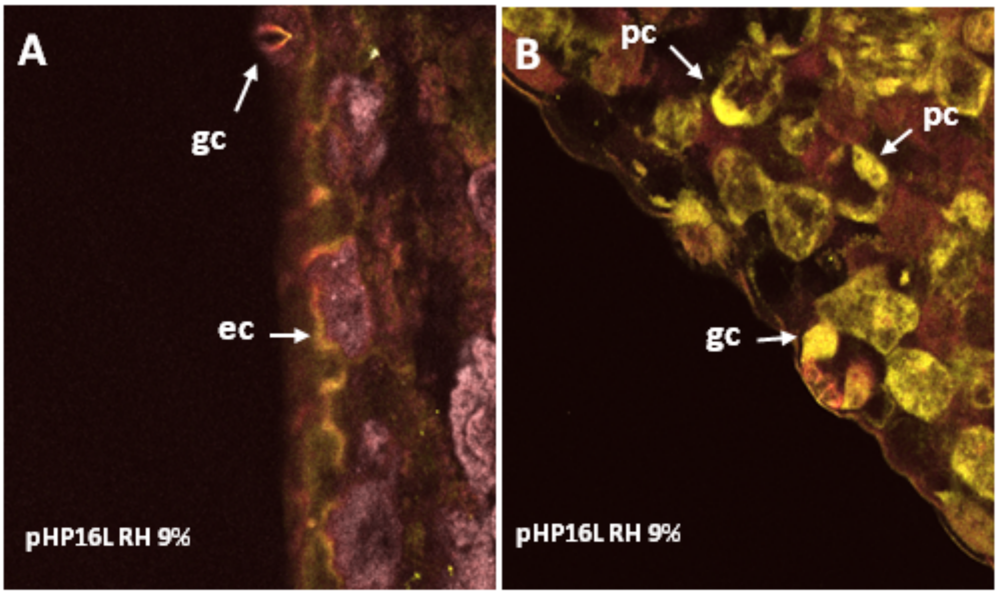

Supplement: Supplementary_Table_S1_Figures_S1_S3 [file erx166_suppl_supplementary_table_s1_figures_s1_s3.pdf]
